# Simultaneous quantification of lead, cadmium and zinc in superficial marine sediments using a carbon-fiber microelectrode modified with bismuth film

**DOI:** 10.1038/s41598-023-47526-6

**Published:** 2023-11-19

**Authors:** Lenys Fernández, Patricio Espinoza-Montero, Mireya Sánchez-Sarango, Diego Bolaños-Méndez, Jocelyne Álvarez-Paguay, Luis Domínguez-Granda, Augusto Rodríguez, Hugo Romero, Alexis Debut, Vladimir Ortiz

**Affiliations:** 1 Escuela de Ciencias Químicas, Pontificia Universidad Católica del Ecuador, 17-01-2184 Quito, Ecuador; 2 Facultad de Ciencias Agropecuarias y Recursos Naturales, Universidad Técnica de Cotopaxi, Latacunga, Ecuador; 3 Facultad de Ciencias Naturales y Matemáticas, Escuela Superior Politécnica del Litoral ESPOL, Campus Gustavo Galindo, Guayaquil, Ecuador; 4 Grupo CAE INIFTA (Instituto de Investigaciones Fisicoquímicas Teóricas y Aplicadas), La Plata, Argentina; 5 Facultad de Ciencias Químicas y de la Salud, Universidad Técnica de Machala, Machala, Ecuador; 6 Centro de Nanociencia y Nanotecnología, Universidad de las Fuerzas Armadas ESPE, 170501 Sangolqui, Ecuador

**Keywords:** Environmental monitoring, Marine chemistry

## Abstract

Marine sediments are a useful environmental assessment matrix as they naturally trap toxic substances of anthropogenic origin and thus have higher concentrations of these than the surrounding water. Therefore, developing methods for the sensitive, accurate, and inexpensive quantification of these substances is important, as the traditional techniques have various disadvantages. The current study evaluated the effectiveness of an in situ bismuth-modified carbon-fiber microelectrode (voltamperometric sensor) to simultaneously detect Pb, Cd, and Zn in marine sediments from Puerto Jeli in El Oro Province, Ecuador. This site is representative of the contamination levels present along the coast in this province. Differential pulse anodic stripping voltammetry was applied, and the resulting linear regression for the metal quantification ranged from 12 to 50 μg mL^−1^, with quantification limits for Pb(II), Cd(II), and Zn(II) of 18.69, 12.55, and 19.29 μg mL^−1^, respectively. Thus, the quantification with the sensor was successful. According to the preliminary results, Cd and Pb values exceeded the permissible limits established by Ecuador (Texto Unificado de la Legislación Secundaria del Ministerio del Ambiente) and the US Environmental Protection Agency, respectively.

## Introduction

Pollutants are substances capable of negatively affecting all ecosystems. This problem remains complex and unsolved for oceans in particular, since humans have been depositing their waste into this type of ecosystem for millennia and continue to do so^1,2^. Particularly in Ecuador, contamination by heavy metals is related to the discharge of effluents from mining extraction processes that release large amounts of these elements into the sea^3,4^. Additionally, water discharge and emissions from residential sectors and other industrial activities have contributed to increasing levels of contamination.

Marine sediments are a main reservoir of heavy metals and are therefore a secondary pollution source in the marine environment^5^. They act as natural xenobiotic traps that accumulate and store substances of anthropic origin^6^. Thus, the transport and suspension of heavy metals in these sediments results in an ecological and biogeochemical imbalance in the ecosystem.

According to López and Hernández^7^, metals such as Pb and Cd have an affinity to hydrogen sulfide groups, –SH, which are commonly present in enzymes and control the speed of metabolic reactions^7,8^; this affinity facilitates the entry of these toxic elements into biological systems. On the other hand, Zn is an essential nutrient and part of the metal–enzyme bond that helps synthesize proteins and nucleic acids and also helps support the immune system, which defends the body from bacteria and viruses^9–11^; however, it can trigger toxic reactions when its concentration levels exceed biological requirements^12^.

Marine sediments are typically deposited as strata, which have different concentrations of contaminants^13,14^. Concentrations are higher in these sediments than in the surrounding water and present little spatial or temporal variation, which is why they are ideal for use as an environmental assessment matrix for this type of ecosystem^15^. In countries such as Greece and South Africa, Cd values of 0.04–998 mg kg^−1^, Pb values of 3–2369 mg kg^−1^, and Zn values of 7–4430 mg kg^−1^ have been found in marine sediments^16^. In Ecuador, Cd values of 0.34–1.54 µg kg^−1^, Pb values of 56.42–20.99 µg kg^−1^, and Zn values of 60–527.17 µg kg^−1^ have been reported^17^, associated with industrial wastewater discharge into the Salado Estuary in Guayaquil.

Most techniques for quantifying heavy metals^18^ have disadvantages, including high cost and the need for well-trained staff, and if analysis is required in situ or in the field, these techniques are far from convenient^19^. Therefore, more versatile analytical methods that allow continuous real-time contamination monitoring are required, in which case sediment evaluation could be an excellent alert tool^20^. Unlike traditional spectroscopic methods, electrochemical techniques for the detection of heavy metals are fast, inexpensive and easy to operate^20–24^.

In the present study, a sensitive electrochemical method applying differential pulse anodic stripping voltammetry (DPASV) was developed to simultaneously quantify Cd, Pb, and Zn using an in situ bismuth (Bi) film-modified carbon-fiber microelectrode (CFµE). This proposed method could be used to determine if the amounts of these metals are within the permissible limits for heavy metals and other contaminants in water and soil established by Ecuadorian law^25^ or the US Environmental Protection Agency (EPA)^26,27^. Puerto Jeli in Ecuador’s El Oro Province was selected as the study site, as it is greatly affected by heavy metal contamination from various anthropogenic activities^28,29^. To the best of our knowledge, little empirical research has been conducted regarding the quantification of heavy metals in sediments in Puerto Jeli.

## Methodology

### Equipment and reagents

This study used a portable potentiostat (CH-Instruments-1230), a three-electrode, one-compartment electrochemical cell, a silver/silver chloride (Ag/AgCl) electrode (CH-Instruments) as the reference electrode, a graphite rod as the counter electrode, and a CFµE as the working electrode. Scanning electron microscope (SEM) images were taken with a Tescan MIRA3 SEM. Flame atomic absorption spectroscopy measurements were taken using a PerkinElmer AAnalyst 600 atomic absorption spectrometer.

The following reagents were used: 98% sulfuric acid (Panreac); 99.8% acetic acid buffer solution (Riedel-de Haen) + 98% sodium acetate (Eka Nobel); Bi(III) standard solutions; Pb(II), Cd(II), and Zn(II) at concentrations of 1000 mg L^−1^ (Spectrum); and 18 mΩ distilled water.

### Working electrode fabrication and activation

Each working electrode was fabricated using a single carbon fiber that was attached to a 10-cm-long copper wire using conductive liquid silver paint. A plastic (PET) tip was then applied and sealed using silicone to isolate the copper wire from the solution, leaving just the fiber exposed to the electrolytic media. A small glass tube was included to protect the exposed copper wire and to give the electrode a pipette appearance. Finally, the carbon-fiber tip was cut to a length of approximately 5 mm.

To activate the working electrode, cyclic voltamperometry was performed in a 0.1 mol L^−1^ H_2_SO_4_ solution as the electrolytic media for 10 consecutive scans (scan rate 100 mV s^−1^).

For the DPASV measurements, an acetate buffer solution (pH 4.5) and Bi(III) (100 μmol L^−1^) were used simultaneously for the in situ formation of the Bi film on the surface of the working electrode, at − 1.3 V. The measurements were carried out without the traditional nitrogen purging.

### Marine sediment sampling

The analyzed marine sediments were collected between November 2021 and March 2022 from Puerto Jeli, located in the Jambelí parish in Santa Rosa Canton in El Oro Province^29^. Santa Rosa Canton is divided into four well-defined zones, and the study area corresponds to approximately 34% of the total area, comprising the entire Jambelí archipelago and the Puerto Jeli urban parish, in which 5% of the population lives^29^. The parish is geographically flat and located 7 m above sea level; the area of the coastal plain is 25,179.79 ha, and that of the tidal plain is 247.61 ha. There is little geological variety: the area mainly comprises marine sediments (24,204.08 ha, [95.19%]) made up of continental and marine materials from the intertidal zone and is influenced by fluvial currents, waves, and tides. Therefore, fine sand and silt high in organic matter and carbonates predominate^29^. Samples of 1 kg of sediment were taken in triplicate at a depth of 3 m (− 3° 6′ 30.36″ N, − 79° 54′ 1.36″ E) and then stored in hermetically sealed polyethylene bags after being washed with 1% HNO_3_. Sediment samples were kept at 4 °C until laboratory analysis was performed.

### Sample treatment

For the treatment of the sample, the Specific Test Procedure Code: CP-PEE-S002 (CESAQ-PUCE) was followed. Samples were homogenized, and foreign objects were removed from the sediment. Next, the quartering technique was applied until approximately 10 g was obtained, which was put in an oven for 1 h at 105 °C^30^. Subsequently, microwave digestion was performed, wherein 0.5 g of the sample was weighed in a vial, 10 mL of nitric acid was added, and the vial was then microwaved at 180 °C for 10 min. The treated sample was allowed to cool, and it was then filtered and transferred to a 25-mL volumetric flask. This same treatment was carried out on the blank (vial with no sample containing only the solvent and reagents). After purging with nitrogen 5 mL of the resulting solution was transferred to the electrochemical cell for analysis.

### Pb, Cd, and Zn quantification using differential pulse anodic stripping voltammetry

The procedure reported by Anandhakumar et al.^23^ was followed, with modifications. For the preconcentration process, a potential of − 1.3 V (vs. Ag/AgCl) was applied for 50 s in a 0.1 mol L^−1^ acetate buffer (pH 4.5) + 100 mol L^−1^ Bi + fixed concentrations of Zn(II), Cd(II), and Pb(II). For the stripping process, an amplitude of 0.25 V; a pulse width of 0.05 s; and a pulse period of 0.1 s were applied. To determine the optimization parameters, calibration plots were created by adding consecutive aliquots to the electrolytic solution to obtain concentrations of 1.06–50.58 µg L^−1^ for each metal from their respective standard reagents. The Zn(II), Cd(II), and Pb(II) content of the sediment samples was determined based on standard calibration plots.

### Quantification of Pb, Cd, and Zn using atomic absorption spectroscopy

Wavelengths of 213.86, 228.80, and 217 nm were used for Zn, Cd, and Pb determination, respectively, in 5 mL of solution resulting from the sample treatment described in the previous section. The instrument settings were an acetylene flow of 10 L min^−1^; a cleaning step between samples lasting 10 s; and the standard calibration mode.

## Results and discussion

### Activation and characterization of the carbon-fiber microelectrode

To improve its surface characteristics, prior to characterization, the CFµE was activated in a H_2_SO_4_ solution, as it has been reported that this type of surface has disordered, porous, and uneven layers of graphite with little effective area and surface activity^31,32,33^. Figure S1 shows the consecutive cyclic voltammetry during the activation of the CFµE in H_2_SO_4_. Ten voltammograms were obtained that showed an absence of signals between potentials − 500 to 1250 mV; signals from water electrolysis were observed at the reduction potential of 750 mV and oxidation at 1500 mV^34^. Previous studies^35^ have shown that this type of treatment for carbonaceous materials causes over-oxidation with the formation of superficial functional groups and charge carriers, which may be compatible with the analyte being studied, thus increasing the electrode’s ionic conductivity and reproducible behaviors.

**Figure 1:**
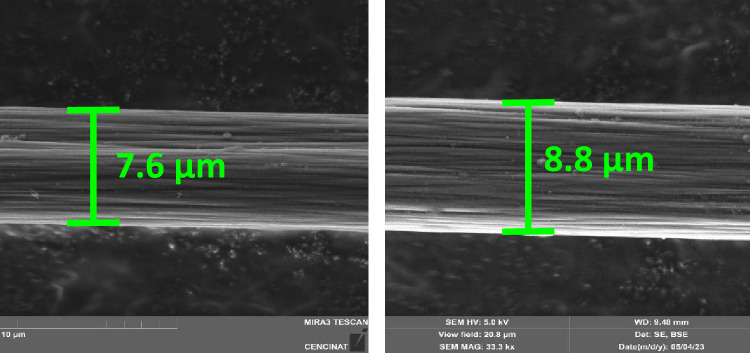
Scanning electron micrographs of an inactivated (**a**) and activated, (**b**) carbon-fiber microelectrode in H_2_SO_4_.

SEM micrographs of an inactivated and activated CFµE in H_2_SO_4_ are shown in Fig. 1. The CFµE before activation had a diameter of 7.6 µm (Fig. 1a), with a pattern of consecutive and horizontally arranged fibers 200 nm in diameter spaced 1.5 nm apart. The SEM micrograph of the CFµE after activation in H_2_SO_4_ (Fig. 1b) shows a much cleaner surface than the inactivated surface, in addition to an increase of up to 8.8 µm in fiber diameter, which reduces the distance between them. After this treatment, the fibrous structure remained well-defined and had less fine fibers.

**Figure 2:**
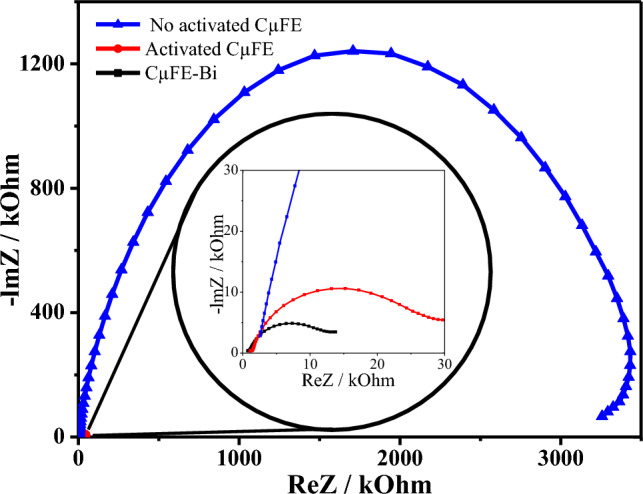
Nyquist plot from EIS for different carbon-fiber microelectrodes.

Figure 2 shows the resulting Nyquist plots for the H_2_SO_4_-activated (2a) and inactivated (2b) CFµEs. A semicircle in the low frequency region is due to charge transfer resistance (Rct) at the electrode/electrolyte interface. According to these results, the semicircle of the H_2_SO_4_-treated electrode has a smaller diameter than that of the inactivated CFµE, indicating a lower Rct resistance.

### Anodic stripping voltammograms of Cd(II), Pb(II), and Zn(II) using differential pulse voltammetry for an in situ carbon-fiber microelectrode modified with Bi film

**Figure 3:**
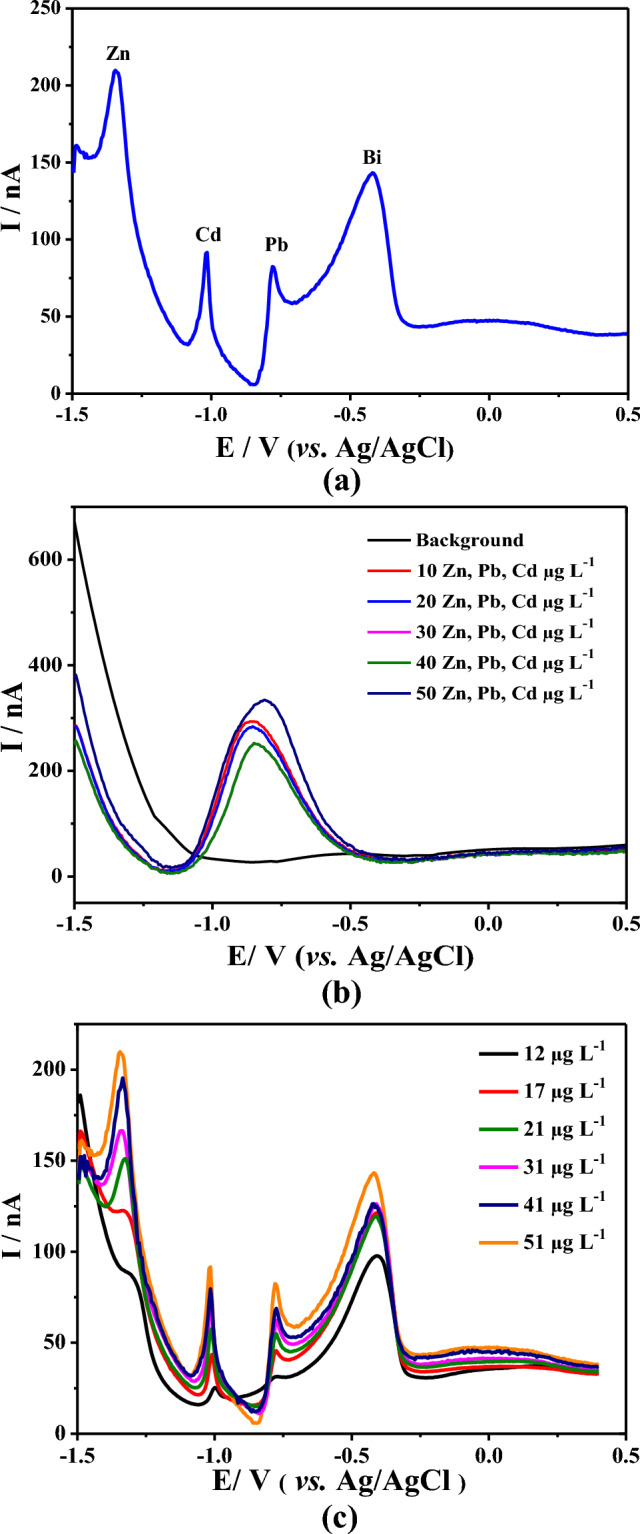
(**a**) Differential pulse voltammetry (DPV) of: (a) carbon-fiber microelectrode (CFμE)-Bi in acetate buffer (pH 4.5) + Bi(III) 100 mol L^−1^ + 50.28 μg mL^−1^ Cd(II), Pb(II), and Zn(II); (**b**) CFμE in acetate buffer (pH 4.5) + Bi(III) 100 mol L^−1^ + 50.28 μg mL^−1^ Cd(II), Pb(I), and Zn(II); (**c**) CFμE-Bi in acetate buffer (pH 4.5) + Bi(III) 100 mol L^−1^ + different metal concentrations.

Mercury is the most widely used metal for DPASV metal detection; however, its toxicity has prompted the development of electrodes based on materials such as gold, platinum, silver, and bismuth^23^. Bismuth is environmentally friendly^36^, which is one reason it is considered a replacement option for mercury, in addition to advantages such as better sensitivity, well-defined signals, and easier metal stripping from the electrode surface. The CFµE used in the present study was modified in situ with a Bi film and used with the three target metals. Figure 3a shows the simultaneous stripping signals using differential pulse voltammetry (DPV) of Zn(II) at − 1.300 mV, Cd(II) at − 1.100 mV, and Pb(II) at − 0.800 mV, with the modified CFµE-Bi. The difference between the potentials obtained for each metal is associated with Bi’s ability to form “fusible” alloys^22^ with different characteristics for each metal^37,38^. A single stripping signal was obtained on an unmodified CFµE (Fig. 3b). With the increase in the metal cation concentrations in the electrolytic medium, the stripping currents on the CFµE-Bi increased (Fig. 3c); this demonstrates the simultaneous sensory activity of the CFµE in the presence of Bi for the three metals.

### Effect of time and preconcentration potential

The effect of the Bi concentration in the electrolytic solution on the metal stripping signals was evaluated using solutions with 70, 100, and 140 µmol L^−1^ Bi. Results showed the 100 μmol L^−1^ Bi concentration had stripping signals with the best definition and separation (data not shown); it was also the concentration for which the CFµE-Bi presented lower Rct than the bare electrode (Fig. 2).

**Figure 4:**
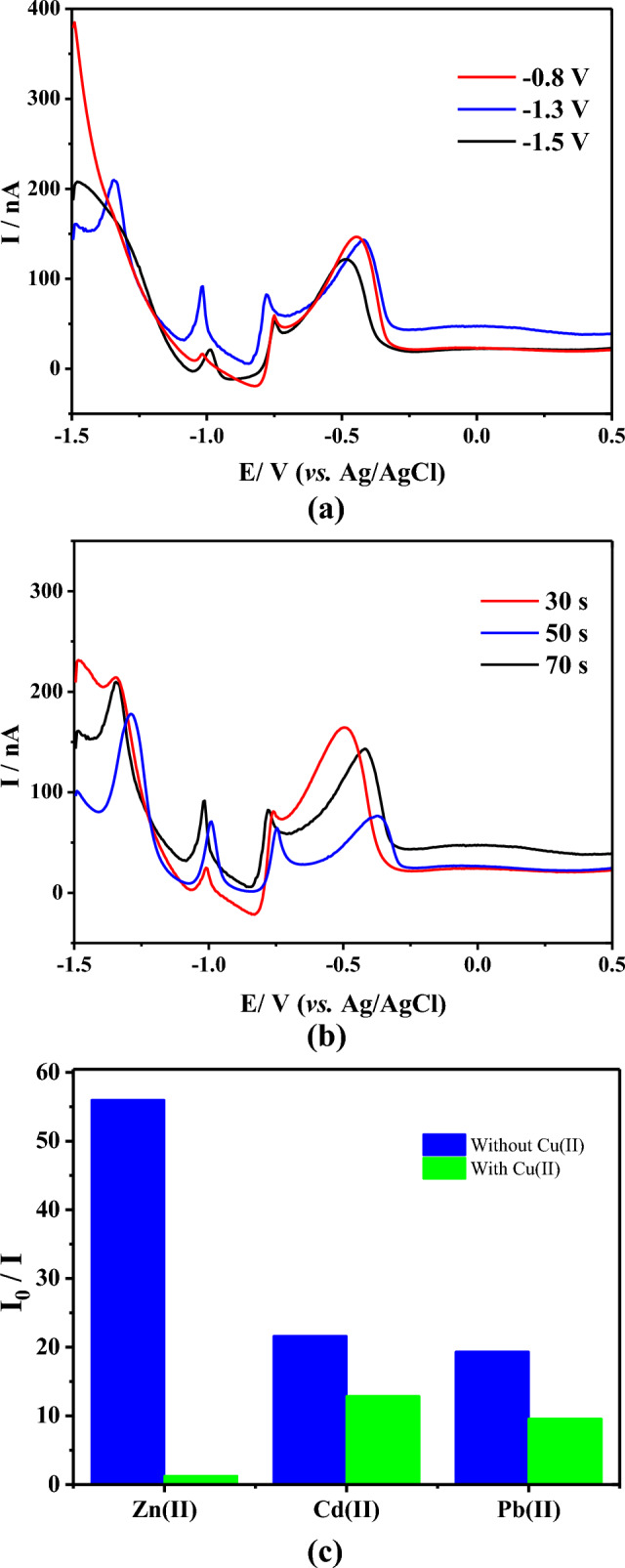
(**a**) Effect of preconcentration potential on metal stripping currents according to differential pulse voltammetry (DPV); (**b**) effect of preconcentration time on metal stripping currents according to DPV; (**c**) effect of interfering Cu(II) ion (100 μg L^−1^) on Zn, Cd, and Pb determination (50 μg mL^−1^ in solution), *n* = 6. Acetate buffer (pH de 4.5) + Bi(III) 100 mol L^−1^.

During the preconcentration stage, potentials between − 0.8 and − 1.5 V were applied to indistinctly reduce the target metal cations on the CFµE surface^5,39^. The potential value to simultaneously detect the three metals was determined to be − 1.3 V, as it was not possible to obtain the Zn signal in the voltammogram at potentials more negative than − 1.5 V and more positive than − 0.8 V (Fig. 4a). In addition, well-defined signals were not achieved for the other two metals. According to the literature^23^, this may because at potentials lower than − 1.5 V, the evolution of hydrogen damages the bimetallic deposits, while at potentials greater than − 0.8 V, the amount of reduced metallic Bi on the µE is insufficient to facilitate the deposition and stripping processes (Figure S1).

It has been reported^23^ that an excessively long preconcentration time for the electrode’s electroactive area can increase the thickness of the Bi film, which destabilizes and then detaches from the electrode surface. Therefore, the preconcentration time was varied in this study to optimize the process, starting with 30 s (Fig. 4b). However, no well-defined or repeatable stripping signals were observed, particularly for Zn, while 50 s allowed for an adequate definition of the desired signals. Although 70 s initially showed encouraging results, deterioration in the responses was observed as the tests proceeded, and the appearance of noise in the voltammograms after each determination suppressed the current signals, which became nonsignificant. On the other hand, the potential displacement observed in the voltammograms at the different preconcentration times indicates that the sensitivity of the metals’ simultaneous stripping signal depends on the thickness of the Bi film formed on the electrode surface; this may be related to the difference in the type of “fusible” alloy that Bi forms with each metal.

### Examination of interference

Previous studies^16,17,39,40^ have indicated that ions such as Fe^2+^, Fe^3+^, Co^3+^, Cu^2+^, Ni^2+^, and Mn^2+^ can interfere with the quantification of the three target metals. The effect of interfering ions was therefore evaluated by comparing the DPASV peak current of Zn(II), Cd(II), and Pb(II) in the absence (I_0_) and presence (I) of interfering metal ions. A decrease of up to 10% of the relative response in the presence of the interfering ion was considered acceptable^39^. Results showed Fe^2+^, Fe^3+^, Co^3+^, Ni^2+^, and Mn^2+^ ions did not significantly affect the (I_0_/I) ratio (data not shown), while Cu^2+^ had the greatest interference. Figure 4c shows the comparison of the DPV stripping current of Zn(II), Cd(II), and Pb(II) in the absence (I_0_) and presence (I) of the Cu(II) ion. In the presence of this interference in the electrolytic medium, the Zn signal practically disappeared, and the Pb and Cd signals decreased, which is associated with the most favorable formation of bimetallic Bi–Cu on the electrode surface.

### Method optimization

**Figure 5:**
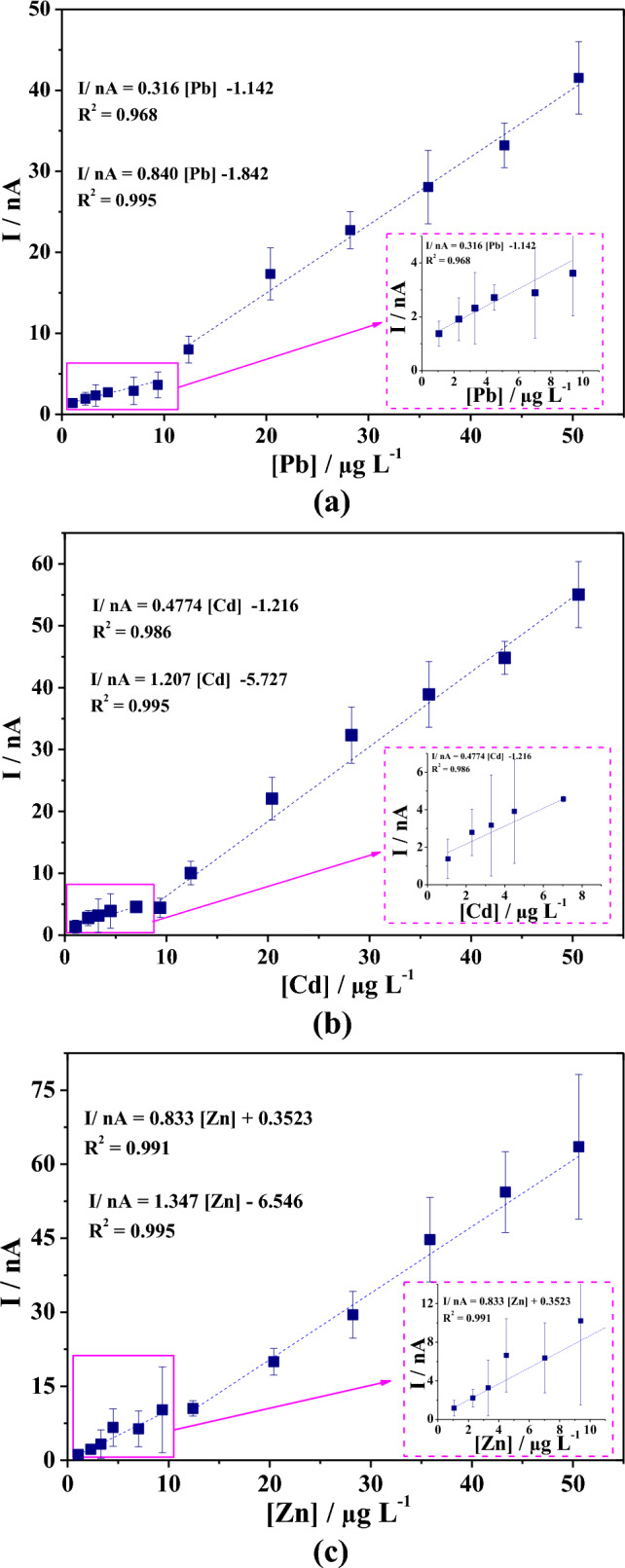
Calibration plots: (**a**) Zn(II), (**b**) Cd(II), and (**c**) Pb(II) in acetate buffer (pH de 4.5) + Bi(III) 100 mol L^−1^ + 50.28 μg mL^−1^ Cd(II), Pb(I), and Zn(II).

The calibration plots in Fig. 5 can be divided into two linear working ranges: low (inserts Fig. 5) and high concentration. Both ranges had acceptable correlation coefficients; however, the sensitivity in the high concentration range was much higher than in the low concentration range. It should be noted that the high concentration range was consistent with previously reported values of metal concentrations in sediments^16,17^, suggesting this method is a good alternative for detecting these trace metals in this type of sample. Based on procedures from the International Union of Pure and Applied Chemistry^41^, the limit of detection (LD) was set as the lowest concentration value, unlike the background current (noise), and can be determined by the Eq. 3xS/m (S = standard deviation and m = slope)^42^. The LD was 0.26 µg mL^−1^ for Pb(II), 0.77 µg mL^−1^ for Cd(II), and 5.79 µg mL^−1^ for Zn(II). The limit of quantification (LQ) was defined as the lowest concentration level that could be reliably quantified by the applied methodology; LQ can be calculated with the Eq. 10xS/m^42^. The LQ was 18.69 µg mL^−1^ for Pb(II), 12.55 µg mL^−1^ for Cd(II), and 19.29 µg mL^−1^ for Zn(II). The interday precision of the measurements was calculated using the relative standard deviation (RSD) of 10 repeated measurements in phosphate buffer solution (pH 4.5) containing the metals in a concentration of 30 µg mL^−1^. The RSD values were 2.76% for Pb(II), 8% for Zn(II), and 3.43% for Cd(II), which suggest excellent repeatability^43^.

### Determination of total Zn, Cd, and Pb in marine sediment samples

Method accuracy was evaluated using a certified reference material for trace element determination in marine sediments (BCR-277R estuarine sediment, trace elements) with certified Zn and Cd concentrations of 178 and 0.61 mg kg^−1^, respectively. The results using flame atomic absorption spectroscopy and the proposed method, DPASV, are presented in Table S1. Statistical analysis using the student's t-test showed no statistically significant difference between the real concentrations and those obtained from both techniques, with a confidence limit of 95%. The recovery percentages for DPASV for Zn and Cd were 99.78% and 114.05%, and the RSD values were 2.3% and 4.4%, respectively. No Pb content was found in the certified sample. These results indicate the feasibility of using the optimized method to quantify Zn, Cd, and Pb simultaneously in real samples of marine sediments.

Table 1 shows the results for the Zn, Cd, and Pb quantification in samples of marine sediments from Puerto Jeli. Flame atomic absorption spectroscopy was used to obtain reference values considered valid for the calculation of the relative error rate. When comparing the results between the two analytical techniques, a relative error percentage less than 10% is considered acceptable^44^. According to the results in Table 1, respective relative error percentages of 8.11%, 8.67%, and 5.29% for Pb, Cd, and Zn were obtained, which indicates that the method had acceptable accuracy.

**Table 1:** Results from marine sediment samples from Puerto Jeli, Ecuador (*n* = 3).

Metal (mg kg^−1^)	FAAS	DPASV	Relative error (%)
Pb	0.37 (± 0.03)	0.34 (± 0.04)	8.11
Cd	0.23 (± 0.04)	0.25 (± 0.03)	8.67
Zn	41.6 (± 0.06)	43.8 (± 0.05)	5.29

*DPASV,*(differential pulse anodic stripping voltammetry); *FAAS* (flame atomic absorption spectroscopy).

This study’s preliminary results indicate the Cd and Pb values exceeded the permissible limits established by the relevant Ecuadorian legislation (Texto Unificado de la Legislación Secundaria del Ministerio del Ambiente) and the US EPA, respectively. To our knowledge, at the time this study was conducted, there was only one study on an electrochemical method for determining heavy metal content in marine sediments. However, the study focused on the simultaneous detection of only Pb and Cd^39^ and used electrodes that require a more sophisticated preparation than that in the present study. Conversely, according to Fernández-Cadena et al.^17^ (in Table 3 of their article), the Pb and Cd concentrations found by the current study were higher than those previously reported for any sediment worldwide, while the Zn concentration was lower than those for the other locations presented in their table.

## Conclusions

The present study optimized a sensitive CFµE-Bi electrochemical sensor to simultaneously quantify Zn, Cd, and Pb in marine sediment samples. The resulting LOQs for Pb(II), Cd(II), and Zn(II) were 18.69 µg mL^−1^, 12.55 µg mL^−1^, and 19.29 µg mL^−1^, respectively. The proposed method was successfully applied to quantify Zn, Cd, and Pb in marine sediment samples from Puerto Jeli, Ecuador, which is affected by metal contamination and for which there is little information regarding heavy metal quantification in sediments. Compared with conventional measurements, the proposed method can be considered an alternative for detecting different heavy metal ions simultaneously. Compared to traditional spectroscopic techniques, electrochemical techniques have the advantage of being fast, inexpensive, easy to operate, and portable.

### Supplementary Information

Supplementary Information.

## Data Availability

The datasets used and/or analyzed during the current study are available from the corresponding author upon reasonable request.
