# Supplementary material for: Simultaneous quantification of lead, cadmium and zinc in superficial marine sediments using a carbon-fiber microelectrode modified with bismuth film
Source: Sci Rep. 2023 Nov 19;13:20232. doi: 10.1038/s41598-023-47526-6 (PMC10658076; doi:10.1038/s41598-023-47526-6)
Supplement: Supplementary file 1 — Supplementary Information. [file 41598_2023_47526_MOESM1_ESM.pdf]

Simultaneous quantification of Pb, Cd and Zn in superficial marine sediments at a carbon fiber microelectrode modified with bismuth film

Lenys Fernández[a]*, Patricio Espinoza-Montero[a]*, Mireya Sánchez-Sarango[a,b], Diego Bolaños-Méndez[a], Jocelyne Álvarez-Paguay[a], Luis Domínguez-Granda[c], Augusto Rodríguez[d], Hugo Romero[e], Alexis Debut[f], Vladimir Ortiz[b]

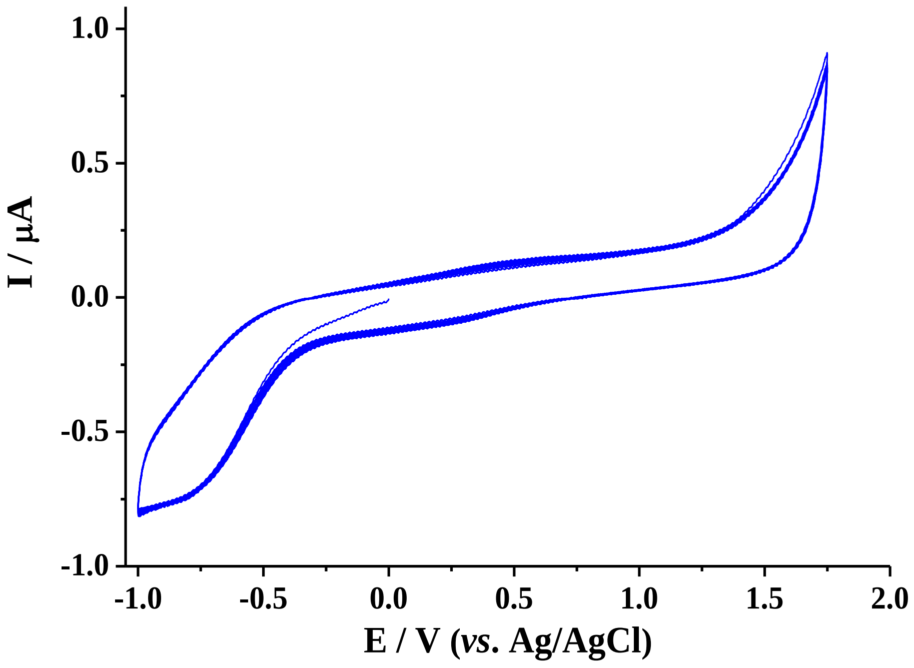

Figure S1. Cyclic voltammetry of activation of a CµFE, in 0.1 M H_2SO_4, 10 consecutive cycles at 100 mv/s

Table S1. Results from the certified material (BCR-277R Estuarine Sediment, trace elements)

	**Pb (mg/kg)**	**Cd (mg/kg)**	**Zn (mg/kg)**
FAAS	-	0.64 (±0.01)	180 (±0.06)
DPASV	-	0.59 (±0.03)	171 (±0.04)
R % by FAAS	-	105.17	102.78
R % by DPASV	-	99.78	114.05
